# Conservative femoral revision using short cementless stems with a tapered rectangular shape for selected Paprosky II–IV bone defects: an average seven-year follow-up

**DOI:** 10.1186/s42836-024-00251-5

**Published:** 2024-06-21

**Authors:** Yicheng Li, Xiaogang Zhang, Baochao Ji, Nuerailijiang Yushan, Wuhuzi Wulamu, Xiaobin Guo, Li Cao

**Affiliations:** Department of Orthopaedics, First Affiliated Hospital of Xinjiang Medical University, 137 South Liyushan Road, Urumqi, Xinjiang 830054 China

**Keywords:** Conservative femoral revision, Short, Cementless stem, Tapered, Rectangular, Bone defect

## Abstract

**Background:**

The use of long stems for severe femoral bone defects is suggested by many scholars, but it is associated with further bone loss, intraoperative fracture, increased surgical trauma, and complications. With better bone retention, simple and quick surgical procedures, and minimal complications, the short cementless stems with a tapered rectangular shape may be an alternative for femoral revision. This study aimed to evaluate the results of this type of stem in treating selected Paprosky II–IV bone defects.

**Methods:**

This retrospective study included 73 patients (76 hips involved) who underwent conservative femoral revision using the short cementless stems with a tapered rectangular shape between January 2012 and December 2020. The preoperative femoral bone defects were identified as follows: 54 cases of type II, 11 cases of type IIIA, 7 cases of type IIIB, and 4 cases of type IV. Indications for revision included aseptic loosening (76.3%) and prosthetic joint infection (23.7%). Six cementless stems with a tapered rectangular shape from three companies were used in all patients. Among them, SLR-Plus, SL-Plus MIA, and Corail stems were employed in most patients (40.8%, 23.7%, and 17.1%, respectively). The average length of these stems measured 171.7 mm (SD 27 mm; 122–215 mm). Radiographic results, Harris hip scores (HHS), complications, and survivorship were analyzed. The follow-up lasted for 7 years on average (range 3–11 years).

**Results:**

The subsidence was observed in three hips (3.9%), and all stems achieved stable bone ingrowth. Proximal femoral bone restoration in the residual osteolytic area was found in 67 hips (88.2%), constant defects in nine hips (11.8%), and increasing defects in 0 cases. There was no evidence of stem fractures and stem loosening in this series. The mean HHS significantly improved from 32 (range 15–50) preoperatively to 82 (range 68–94) at the last follow-up (*t* = − 36.297, *P* < 0.001). Five hips developed prosthesis-related complications, including three infection and two dislocation cases. The mean 5- and 10-year revision-free survivorships for any revision or removal of an implant and reoperation for any reason were 94.6% and 93.3%, respectively. Both mean 5- and 10-year revision-free survivorships for aseptic femoral loosening were 100%.

**Conclusion:**

Conservative femoral revision using short cementless stems with a tapered rectangular shape can provide favorable radiographic outcomes, joint function, and mid-term survivorship with minimal complications. Of note, a sclerotic proximal femoral bone shell with continued and intact structure and enough support strength is the indication for using these stems.

**Graphical Abstract:**

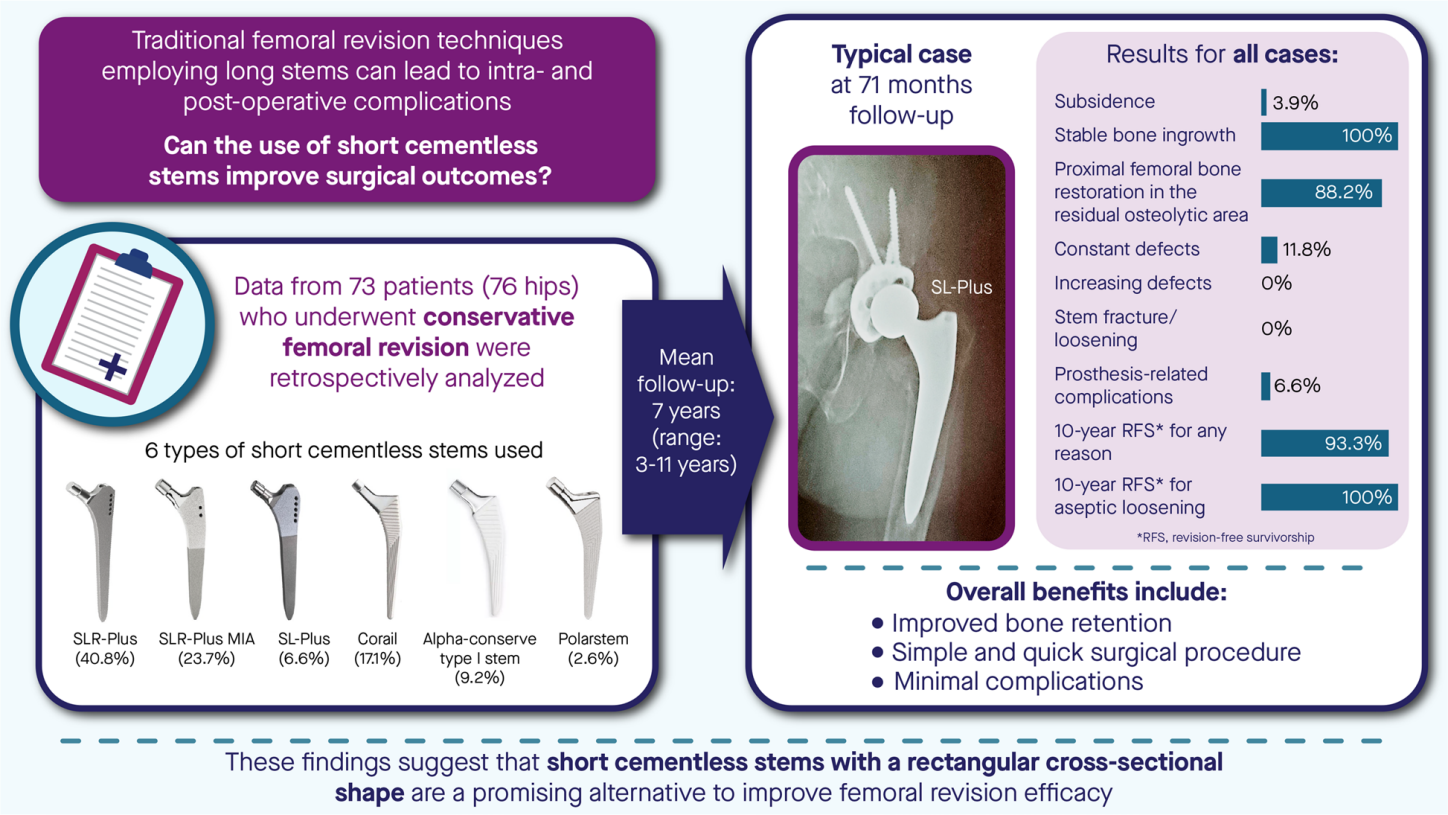

## Introduction

With the rate of total hip arthroplasty (THA) mounting rapidly, the rate of revision THA also has been on the rise annually, the number of procedures being projected to increase from 40,800 in 2005 to 96,700 in 2030 [1]. A challenge posed by the femoral revision is various degrees of bone defects, particularly Paprosky II–IV [2], which result in insufficient support for the prosthesis, thus, leading to subsidence, loosening, and other serious complications [3–6].

A variety of components have been developed to manage severe femoral bone loss, mainly including extensively coated stems [7] and modular fluted tapered stems [8, 9]. However, several complications, including substantial stress shielding, fatigue fractures, and corrosion at several junctions, have also been reported to be associated with these stems [7–9]. Moreover, compared with Europeans and Americans, Asians have relatively shorter length and larger anterior bowing of the femur [10]; hence, long stems for revision are more likely to cause periprosthetic fractures and thigh pain. Considering the younger age of revision patients, fracture and infection can be catastrophic for them due to the residual bone mass failing to withstand future revisions.

The use of cementless stems with a tapered rectangular cross-sectional shape not only achieved excellent long-term results in the primary replacement [11] but also attained acceptable outcomes in the hip revision with limited bone defects [12–14]. This type of component is designed to accommodate proximally located bony defects and provide enhanced load transfer from the proximal to the distal femur [12], particularly providing initial secure axial positioning and excellent anti-rotation stability [13]. Furthermore, the operative technique involving these stems is simple, which shortens the operation time, reduces intraoperative bleeding, and, more importantly, preserves more host bone mass, leaving the opportunity for possible re-revision [14]. However, to our knowledge, reports are still scanty on the application of these stems to address severe femoral defects.

This retrospective study reviewed a cohort of consecutive patients with Paprosky II-IV femoral bone loss who had been treated with short cementless stems with a tapered rectangular shape, and the purpose was to use a conservative femoral revision strategy to resolve complex femoral bone defects and reduce the risk of intraoperative fractures and postoperative complications. This study aimed to answer 4 questions: (1) What are the surgical indications for using this type of stems? (2) Will joint function improve in terms of the Harris Hip Score (HSS) after conservative femoral revision? (3) What are the postoperative complications? and (4) What is the medium- and long-term survival rate of these stems?

## Patients and methods

### Demographics of patients

The medical records of patients who had undergone revision hip arthroplasty by an experienced orthopedic surgeon (LC) were reviewed between January 2012 and December 2020. Patients were included if they underwent revision THA using the short cementless stems with a tapered rectangular shape for selected Paprosky II–IV bone defects. Patients receiving follow-up < 2 years and having incomplete data were excluded. Finally, 73 patients (76 hips) were included. The preoperative femoral bone defects were identified according to Paprosky classification [2] as follows: 54 cases of type II, 11 cases of type IIIA, 7 cases of type IIIB, and 4 cases of type IV. The reasons for femoral stem revision included aseptic loosening (*n* = 58; 74.2%) and prosthetic joint infection (PJI) (*n* = 18; 23.7%). Replacement of a cemented stem was performed in 8 revised hips, and combined acetabular revision was performed in 66 hips. Demographic data of all cases were strictly recorded (Tables 1 and 2).

**Table 1:** Demographic data

Demographic	Aseptic loosening	PJI
Total	58	18
Male, *n*	27	12
Mean age, years (SD)	58 (12)	58 (12)
Mean body mass index, kg/m^2^ (SD)	24 (5)	25 (4)
ASA classification, *n*		
I	13	2
II	39	11
III	4	5
IV	2	N/A
History of blood transfusion, *n*	21	4
Sinus tract, *n*	N/A	5
No. of previous joint surgery, *n*		
1	9	4
2	1	2
≥ 3	N/A	2
Paprosky classification of femoral bone defects, *n*		
II	42	12
IIIA	7	4
IIIB	5	2
IV	4	N/A

*PJI* Periprosthetic joint infection, *ASA* American Society of Anesthesiologists, *N/A* Not applicable

**Table 2:** Surgery-related characteristics

Parameters	Aseptic loosening	PJI
Only stem revision, *n*	7	3
Operation duration, min (SD)	101 (22)	97 (12)
Blood transfusion volume, mL (SD)	520 (160)	460 (120)
Total hip revision, *n*	51	15
Operation duration, min (SD)	124 (49)	125 (50)
Blood transfusion volume, mL (SD)	780 (500)	760 (500)
Cement stem before revision, *n*	6	1
Extended trochanteric osteotomy, *n*	8	3
Paprosky classification of acetabular bone defects, *n*		
I	17	8
IIA	8	2
IIB	6	N/A
IIC	10	3
IIIA	5	N/A
IIIB	5	2
Reinforced devices		
Augments	4	N/A
Cage	3	N/A
Multicup	9	4

*PJI* Periprosthetic joint infection, *N/A* Not applicable

### Detailed information of stems

**Fig. 1:**
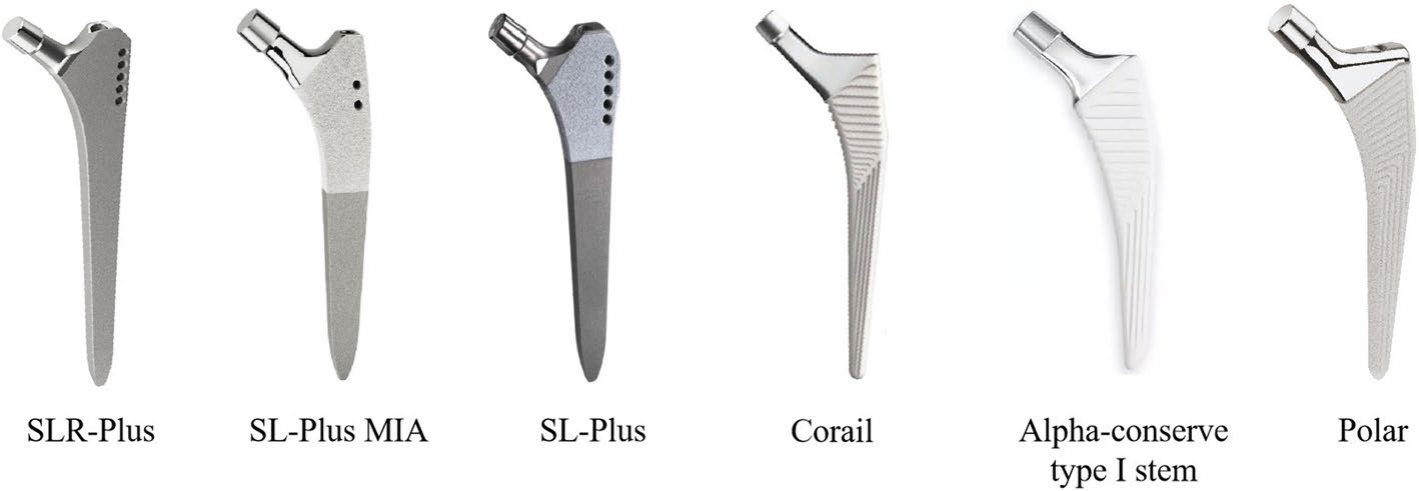
A photograph shows six cementless stems with a tapered rectangular shape from three companies

**Fig. 2:**
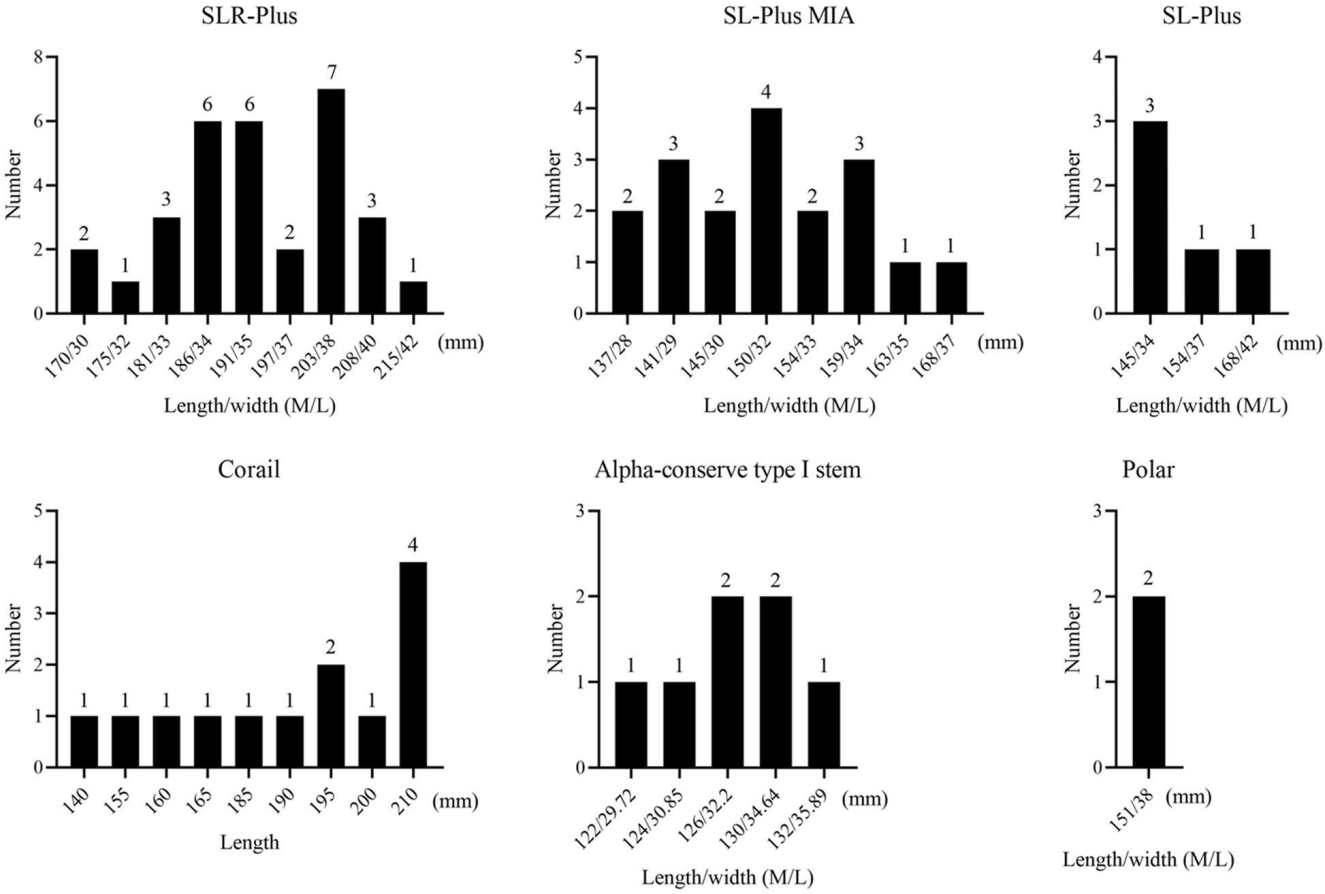
A photograph shows the specific application information of six stems. M/L, medial and lateral transverse diameter

**Fig. 3:**
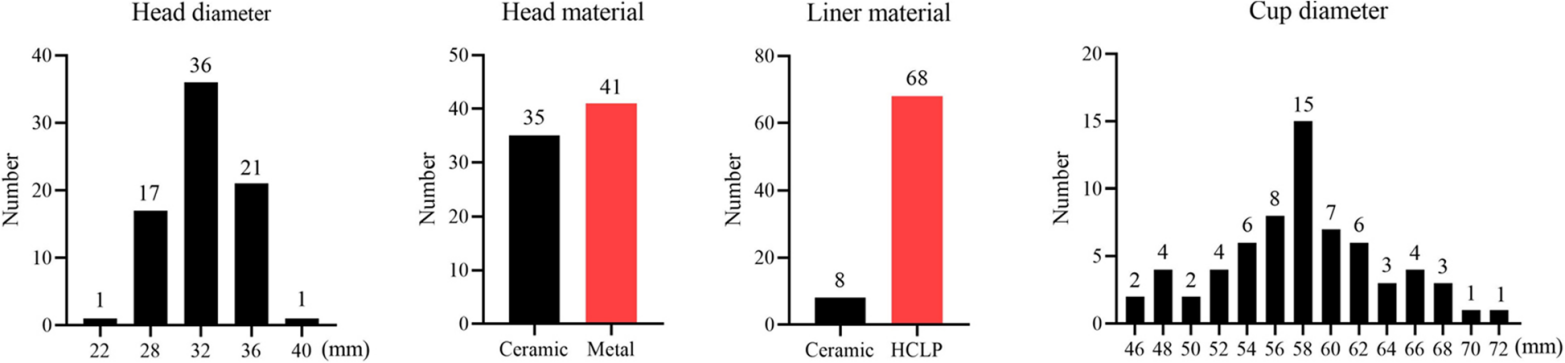
A photograph shows the diameters and materials of the head, liner, and cups used in this study

Six cementless stems with a tapered rectangular shape from three companies were used in all patients (Fig. 1). Among them, SLR-Plus, SL-Plus MIA, and Corail stems were used in most patients (40.8%, 23.7%, and 17.1%, respectively). SLR-Plus, SL-Plus, and SL-Plus MIA stems are made of a titanium-6 aluminum-7 niobium (Ti-6Al-7Nb) alloy, and the first two stems have a grit-blasted surface, while the last stem has a hydroxyapatite-coating. Corail, Polar, and Alpha-conserve type I stems are made of titanium alloy and have hydroxyapatite coating (Table 3). The data of different stems are detailed in Fig. 2, and the average length of the stems was 171.7 mm (SD 27 mm; 122–215 mm). The heads of the following diameters were used: 22 mm in 1 procedure (1.3%), 28 mm in 17 procedures (22.4%), 32 mm in 36 procedures (47.4%), 36 mm in 21 procedures (27.6%), and 40 mm in 1 procedure (1.3%). A metal head and high crosslinked polyethylene liner were used in most patients (53.9% and 89.5%, respectively). The top three diameters of the cups used were as follows: 58 mm in 15 cases (22.7%), 56 mm in 8 cases (12.1%), and 60 mm in 7 cases (10.6%) (Fig. 3).

**Table 3:** Specific information of femoral stems used in this study

Classification of femoral stems	Number	Percent	Coating	Material
SLR-Plus; Smith & Nephew (Memphis, TN, USA)	31	40.8%	Grit-blasted surface	Ti-6Al-7Nb alloy
SL-Plus MIA; Smith & Nephew (Memphis, TN, USA)	18	23.7%	Hydroxyapatite	Ti-6Al-7Nb alloy
SL-Plus; Smith & Nephew (Memphis, TN, USA)	5	6.6%	Grit-blasted surface	Ti-6Al-7Nb alloy
Corail; DePuy Synthes (Warsaw, IN, USA)	13	17.1%	Hydroxyapatite	Ti alloy
Alpha-conserve type I stem; Wego Orhto (Shandong, China)	7	9.2%	Hydroxyapatite	Ti alloy
Polarstem; Smith & Nephew (Memphis, TN, USA)	2	2.6%	Hydroxyapatite	Ti-6Al-4V alloy

### Surgical technique

A posterior approach was used in all cases. Tissue and fluid specimens for histological analysis and microbial culture were routinely harvested. After joint dislocation, an extractor was employed to try to pull out the stem. If this procedure was difficult, the ETO was performed. According to the preoperative radiographs and intraoperative findings, the femoral bone defect was checked again. Then, the stability of the acetabular cup was also evaluated by using a scalpel to pass through the interface between the implant and the bone or Allis forceps to tug the implant. If this procedure was uneventful, the component was considered reliably fixed. If the acetabular cup loosened, the bone defect was also assessed after prosthesis removal. According to the degree of acetabular bone defect, a new prosthesis was implanted. Then, the remaining femoral canal was prepared with reamers under direct vision. If the intraoperatively determined bone conditions indicated that the shaft had the risk of fracture, the use of a titanium band cerclage was recommended to protect the integrity of the bone shell before starting the preoperative process with the rasp. Decisions about the length and size of the components and limb length were made with the assistance of fluoroscopy. After repeated confirmation of joint stability, non-absorbable sutures were used to attach the remaining abductors to the lateral part of the greater tuberosity, and then, the surgical incision was sutured layer by layer.

### Medication regimen

All patients were intravenously given 1.5 g tranexamic acid within 1 h before surgery and 0.5 g tranexamic acid (q12h) after operation for 3 days. After surgery, peroxib sodium (40 mg, q12h) was administered intravenously for 7 days, followed by oral celecoxib (120 mg, qd) for 1 month. For aseptic loosening, patients received intravenous 1.5 g cefuroxime sodium within 1 h before surgery and every 12 h for 24 h after surgery. For PJI, patients received intravenous sensitive antibiotics within 1 h before surgery and intravenous and intra-articular injection of sensitive antibiotics after surgery, which was consistent with our previous study [15].

### Postoperative rehabilitation

In the cases of aseptic loosening, patients who had Paprosky II femoral bone defect and did not use the tantalum block, cup, or cage for acetabular bone defect, were allowed to gradually engage in weight-bearing after surgery, but with a walker or crutches, for 6–8 weeks. If patients had Paprosky III–IV femoral bone defects or received tantalum cup or cage for acetabular bone defect, they had to be bedridden for 4–6 weeks following the operation, and then, a walker or crutches were used to assist gradual weight-bearing for 4–6 weeks. Quadriceps isometric contraction and ankle pump training were required for every patient after the operation, and the intensity of training depended on each patient’s conditions. In the cases of PJI, the patient was strictly confined to bed for 2 weeks, during which antibiotics were administered by intravenous and intra-articular injection for anti-infective treatment.

### Follow-up and assessments

Two orthopedic surgeons who did not participate in surgery reviewed patients at 1, 3, 6, and 12 months postoperatively and then at yearly intervals. The evaluation included radiological measurements, functional scores, complications, and survival. When patients could not return for follow-up, telephone and mail surveys were used to obtain information.

The axial stem subsidence, defined as the distance between the lesser trochanter and the tip of the prosthesis, was measured using bone-prosthetic landmarks [16]. The measured subsidence was defined as the difference between immediate postoperative radiographs and radiographs at a final follow-up. The femoral implant stability was radiographically classified, according to the criteria of Engh et al., as stable through osseointegration, fibrous stable, and unstable [17]. Proximal bone restoration in residual osteolytic areas was subjectively classified as osseous restoration, constant defects, or increasing defects, according to the criteria of Böhm et al. [18]. The Harris Hip Score (HHS) was used for the comparison of joint function before and after surgery. Prosthesis-related complications were assessed, including infection, dislocation, loosening, fracture, and thigh pain. The Kaplan-Meier method was used to analyze the survival of all patients.

### Statistical analysis

The Mann–Whitney U test was performed to examine any differences between pre- and postoperative HHS. All statistical analyses were performed using SPSS v. 18.0 software (SPSS, USA) with statistical significance set at a *P*-value < 0.05.

## Results

**Fig. 4:**
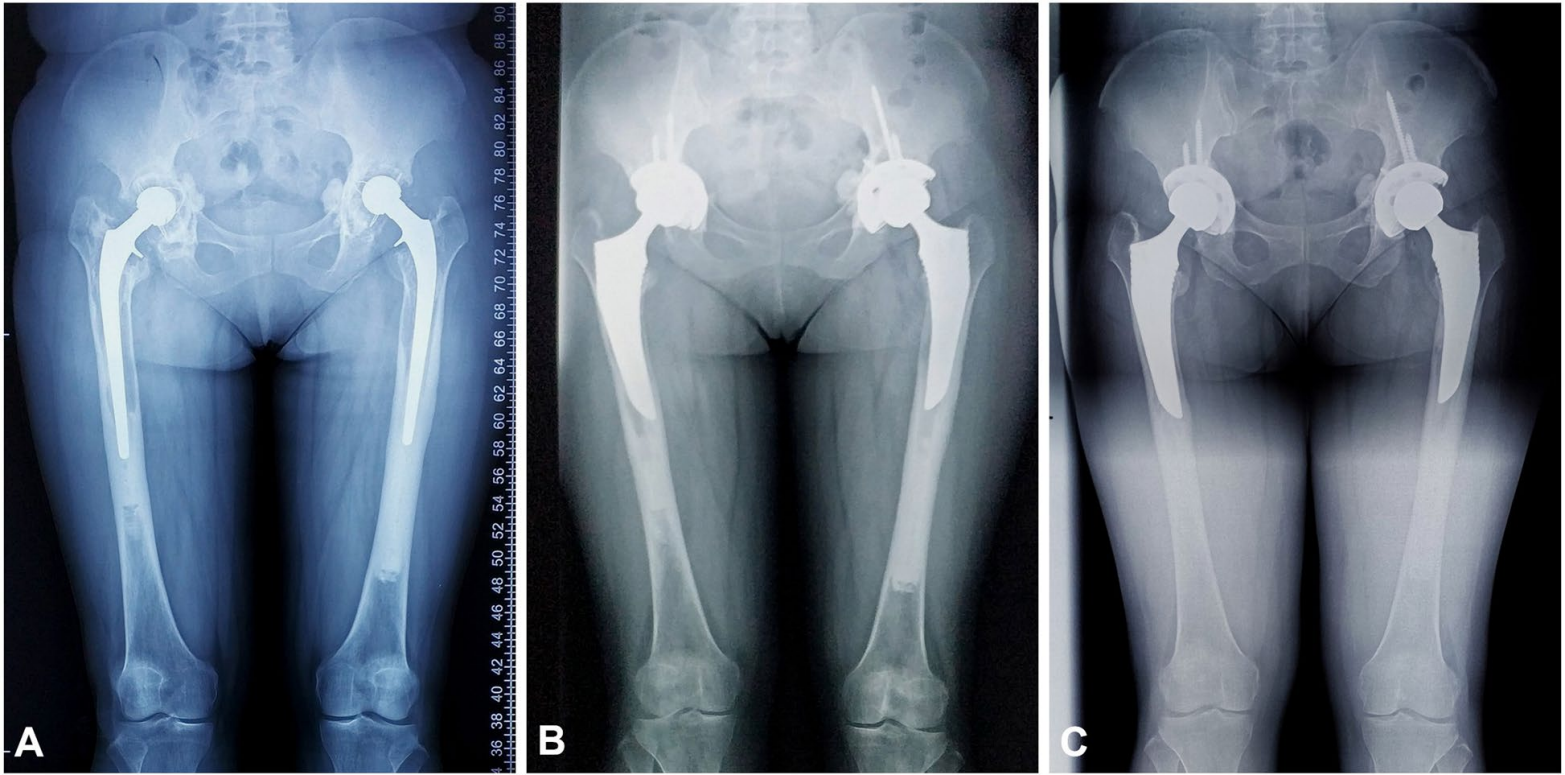
Radiographs show **A** proximal femoral bone defects (Paprosky IIIB in the right hip and IIIA in the left hip) in a 45-year-old female patient with aseptic loosening, **B**, **C** the fixed cementless alpha-conserve type I stem without radiolucent lines, osteolysis, or migration at 3- and 60-month follow-up

**Fig. 5:**
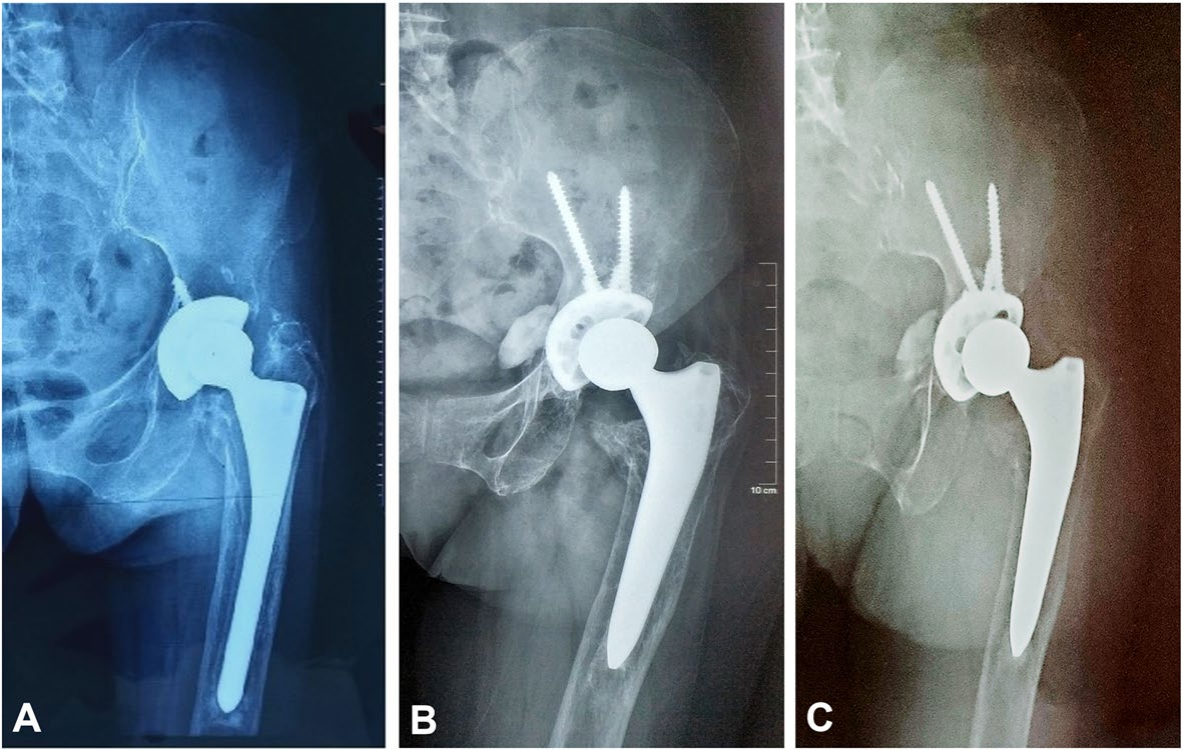
Radiographs show **A** proximal femoral bone defect (Paprosky IV) in a 68-year-old female patient with aseptic loosening, **B**, **C** the fixed cementless SL-Plus MIA stem without osteolysis, subsidence, or loosening at 15- and 71-month follow-up

All cases were followed for an average of 7 years (range 3–11 years). The subsidence was observed in three hips (3.9%), in which one hip subsided for 3 mm (type IIIA), one hip subsided for 5 mm (type IIIB), and the other hip subsided for 9 mm (type IV). All stems achieved stable bone ingrowth. Proximal femoral bone restoration in the residual osteolytic area was observed in 67 hips (88.2%), constant defects were found in nine hips (11.8%), and no increasing defects appeared. Union was seen radiographically in all cases of ETO. There was no evidence of stem fractures and stem loosening, including radiolucent lines, osteolysis, migration, and distal pedestal formation, in this series. Typical cases are shown in Figs. 4 and 5.

The mean HHS improved significantly from 32 (15–50) preoperatively to 82 (68–94) at the last follow-up (*t* = − 36.297, *P* < 0.001) for all patients.

A total of five patients had prosthesis-related complications. The most common complication was infection, which occurred in three patients. After one-stage revision, one case underwent debridement and exclusion because of poor infection control. A new prosthesis was re-implanted 3 months after surgery, and open reduction for dislocation was performed 6 months later. The other two cases developed PJI 2 and 3 years after surgery, respectively. Of them, one underwent debridement, and the other received one-stage revision. Dislocation occurred in two patients 1 month after surgery. One case was treated with open reduction, and another case was again dislocated 1 year after open reduction and had to undergo revision THA.

**Fig. 6:**
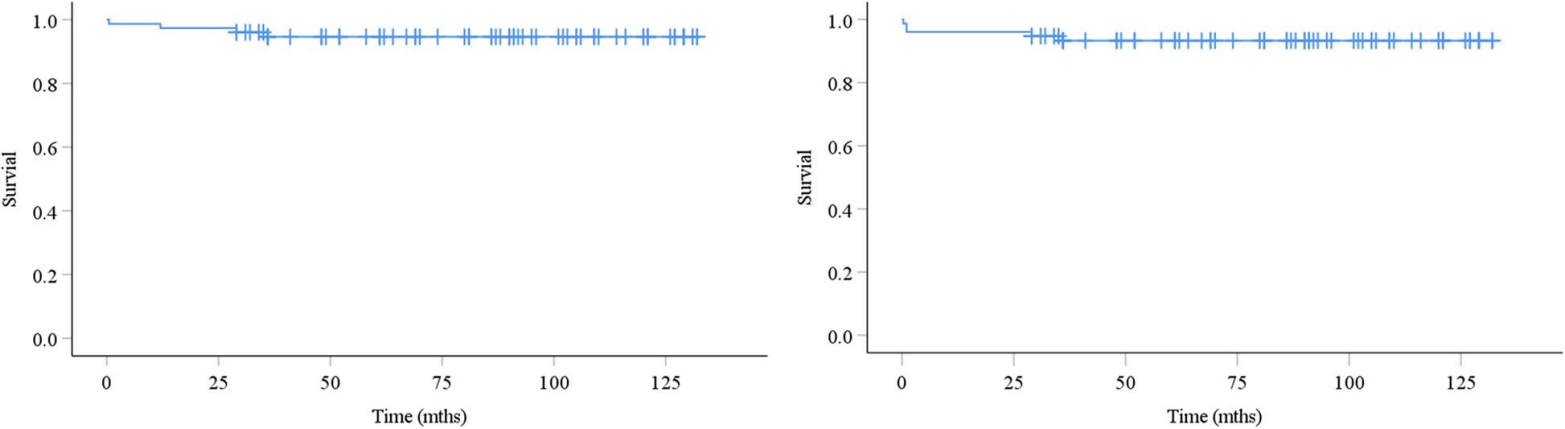
Kaplan-Meier survivorship free of any revision or removal of an implant or reoperation for any reason

Both mean 5- and 10-year revision-free survivorships for any revision or removal of an implant and reoperation for any reason were 94.6% and 93.3%, respectively (Fig. 6). Both mean 5- and 10-year revision-free survivorships for aseptic femoral loosening were 100%.

## Discussion

Many scholars prefer to use long, large-diameter, versatile stems to obtain favorable outcomes in the short- and mid-term periods, but related severe complications also emerge [19–21]. In these situations, relatively shorter cementless stems with specific morphological features may be an alternative for femoral revision. Although stem designs have evolved over the past 30 years for a variety of reasons, this type of stem still has its value in revision or complex primary settings [11].

**Fig. 7:**
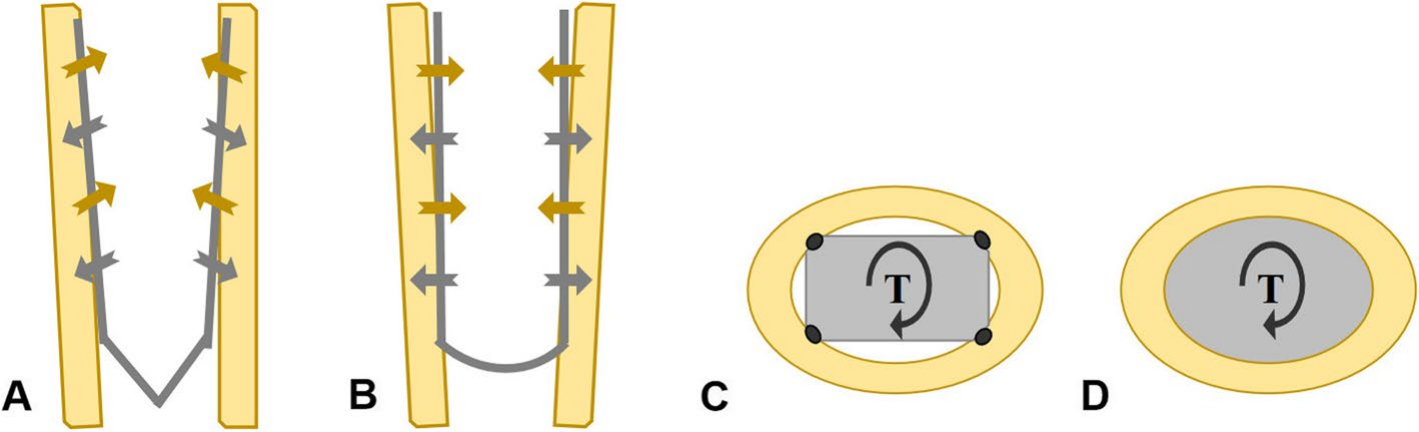
The fixation of two types of stems. Compared with the cylindrical stem fixed at the distal femur through surface contact (**B**, **D**), the taper stem obtains further endosteal engagement and a resultant increase in circumferential compression of the implant (**A**) as well as greater resistance to rotation by the way of point fixation (**C**)

Chang et al. [22] reported that the rectangular tapered cementless stem could achieve excellent initial stability in any morphologic shape of the femur due to its two main features. On the one hand, the rectangular cross-sectional shape provides four-point fixation along the four corners within the femoral canal, contributing to rotational stability. On the other hand, the dual-tapered shape allows for further endosteal engagement, greater stem-diaphyseal diametric mismatch, and a resultant increase in circumferential compression of the implant, ensuring axial stability (Fig. 7). Although our study included stems from different companies, their design concepts were consistent with the above characteristics. Compared with long cylindrical stems and tapered fluted stems [2, 23, 24], the stems used in this study were shorter in length but greater in medial and lateral transverse diameters, which allows the stem to be engaged at the canal of proximal femur with intact cortex without estimating the extent of femoral bone defect. Moreover, the tapered stems only needed a minimum intact segment of 1.5–2.5 cm to obtain adequate initial fixation stability [25], which was shorter than 5–7 cm required by cylindrical stems [26]. Russell et al. [27] revealed that average loads to produce 150-μm displacement or failure were higher for tapered stems than for cylindrical stems.

The indication for a long cylindrical stem fixed distally in patients with severe bone defects [28, 29] does not apply to the short tapered rectangular stem, we believe that the selection principles of the latter should conform to the following: a continued and intact proximal femoral bone shell and sclerotic proximal femoral bone strong enough to support the stems. Moreover, three points should also be emphasized: (1) the rasping of the femur medullar cavity should be gentle, not like during the initial THA, and a cable should be used if necessary; (2) residual bone cement in the distal medullar cavity of the femur does not need to be forcibly removed, to avoid extra bone loss and fracture; (3) early postoperative loading of patients should be avoided. If the aforementioned conditions are satisfied, the complicated surgical procedure will become easier and faster, consequently resulting in less bleeding and minimal complications.

A study reported a high rate of subsidence (16.1%–23.6%) in Paprosky I–IV femoral bone loss treated by modular and monoblock-tapered fluted titanium tapered stems [30], the rate being considerably higher than our results (3.9%). In our opinion, the disparity could be ascribed to (1) although both were tapered stems, the stem with wedge-shaped geometry used in this study was “point-fixed”, while the others used in previous studies were “face-fixed”, which impairs the endoosseous blood supply and affects bone remodeling; (2) sufficient press-fit is beneficial for the dual-tapered cementless stem to accomplish excellent initial stability; (3) our patients with severe bone defects were instructed to stay long in bed, which is critical since it allowed enough time for osseointegration. The latter two points were also supported by Zhang et al. [25], who revealed that insufficient press-fit and premature weight bearing were the main factors responsible for subsidence. Although radiographic subsidence was observed in three hips, none of them had clinical failure requiring reoperation, due to “secondary stability”, meaning the tapered stem with a larger diameter of the proximal portion can achieve incremental fixation with stem subsidence [27].

The osseointegration rate in our series was up to 100% in contrast to 83.5% reported with the Wagner SL revision component [31] and 56% with the modular ExtrêmeTM (Mark I) stem [32]. This finding is supported by a previous report [22], revealing that the “fit without fill” technique of tapered rectangular stems is conducive to preserving the blood supply of the endosteum, which can, in turn, promote bone regeneration, thus, finally restoring femoral bone stock and achieving the two-stage biological fixation. Both excellent bone ingrowth and long-term stability are important factors in reducing the incidence of thigh pain, which explains our results of no cases having thigh pain. Additionally, the stiffness mismatch between bone and prosthesis is also responsible for thigh pain [33]. Compared to the cobalt-chromium alloy, the elastic modulus of titanium alloy is closer to the femur shaft [34, 35], which is another advantage of this type of stem in easing thigh pain.

Regarding the studies on short tapered cementless stems for femoral revision (Table 4), the HHS in the current study was similar to those reported by Uriarte et al. [14] and Chang et al. [22], showing that final results were ≥ 80 points. However, Korovessis et al. [12] and Wang et al. [36] achieved 65.5 and 68.1 points at the last follow-up, respectively. The main reasons were different degrees of femoral defects and lower preoperative HHS in the latter. Furthermore, as PJI was treated by a one-stage revision in this cohort, the soft tissue around the hip could instantly obtain enough tension after surgery, and the combined systematic rehabilitation training compensated the disadvantages of long-term bed rest, consequently improving joint function and accelerating the rehabilitation process [37–40].

**Table 4:** Reports on the femoral revision using cementless stems with a tapered rectangular shape

Study	Year	Hips (*n*)	Age (years)	Indications (*n*)	Classification of bone defects (*n*)	Follow-up (years)	Survivorship (%)	HHS
Korovessis et al. [7]	2009	59	69	Aseptic loosening (47) Dislocation (2) Periprosthetic fracture (6) Infection (4)	AAOSC: II 45, III 6, IV 1, V 1, VI 6	8.3	94 (removal for any cause) 95 (removal for aseptic loosening)	65.5
Uriarte et al. [9]	2019	66	70.5	Aseptic loosening (38) Periprosthetic fracture (3) Infection (21) Cup revision (1) Instability (1) Adverse reaction to metal debris (2)	Paprosky: I 25, II 31, IIIA 8, IIIB 2	4.1	100 (removal for aseptic loosening) 95.5 (revision for any reason)	83
Chang et al. [17]	2011	48	66.5	Aseptic loosening (26) Periprosthetic fracture (2) Infection (2) Cup revision (18)	Paprosky: I 19, II 14, IIIA 4	5.6	98 (removal for any cause)	91.6
Wang et al. [31]	2019	75	54.7	Aseptic loosening (69) Periprosthetic fracture (4) Infection (2)	Paprosky: I 4, II 38, IIIA 19, IIIB 13, IV 1	14	90 (removal for any reason)	68.1
Present study	2023	76	57	Aseptic loosening (58) Infection (18)	Paprosky: II 54, IIIA 11, IIIB 7, IV 4	6.6	94.6 (removal for any cause) 93.3 (reoperation for any reason)	82

*AAOSC* American Academy of Orthopaedic Surgeons Committee, *N/A* Not applicable

Our study was subject to several limitations. First, this was a retrospective study with a small sample size, which potentially introduced bias. Second, the advanced age and poor health of patients who presented with significant bone loss limited complete follow-up. Third, this study lacked a control group. Therefore, subsequent studies should focus on the comparison of efficacy between the stems used in this study and other tapered stems in the long-term follow-up.

## Conclusions

Using the short cementless stems with a tapered rectangular shape, conservative femoral revision could obtain promising radiographic results, such as low subsidence rate and excellent osseointegration, rapid functional recovery, and few complications, as well as favorable survivorship in the setting of selected Paprosky II–IV bone defects at a mean seven-year follow-up. Notably, the indication for selecting these stems should be strictly followed to ensure an excellent outcome.

## Data Availability

This study was carried out in the First Affiliated Hospital of Xinjiang Medical University (137 South LiYuShan Road, Urumqi, Xinjiang, China). The datasets used and/or analyzed during the current study are available from the corresponding author on reasonable request.

## Abbreviations

THA: Total hip arthroplasty
HSS: Harris hip score
